# Multi-Modality Image Fusion and Object Detection Based on Semantic Information

**DOI:** 10.3390/e25050718

**Published:** 2023-04-26

**Authors:** Yong Liu, Xin Zhou, Wei Zhong

**Affiliations:** 1 School of Software Technology, Dalian University of Technology, Dalian 116620, China; 2 International School of Information Science & Engineering, Dalian University of Technology, Dalian 116620, China

**Keywords:** computer vision, deep learning, image fusion, object detection, neural architecture search

## Abstract

Infrared and visible image fusion (IVIF) aims to provide informative images by combining complementary information from different sensors. Existing IVIF methods based on deep learning focus on strengthening the network with increasing depth but often ignore the importance of transmission characteristics, resulting in the degradation of important information. In addition, while many methods use various loss functions or fusion rules to retain complementary features of both modes, the fusion results often retain redundant or even invalid information.In order to accurately extract the effective information from both infrared images and visible light images without omission or redundancy, and to better serve downstream tasks such as target detection with the fused image, we propose a multi-level structure search attention fusion network based on semantic information guidance, which realizes the fusion of infrared and visible images in an end-to-end way. Our network has two main contributions: the use of neural architecture search (NAS) and the newly designed multilevel adaptive attention module (MAAB). These methods enable our network to retain the typical characteristics of the two modes while removing useless information for the detection task in the fusion results. In addition, our loss function and joint training method can establish a reliable relationship between the fusion network and subsequent detection tasks. Extensive experiments on the new dataset (M3FD) show that our fusion method has achieved advanced performance in both subjective and objective evaluations, and the mAP in the object detection task is improved by 0.5% compared to the second-best method (FusionGAN).

## 1. Research Background and Introduction

A single sensor has its limitations, and it is challenging to create a thorough, credible, and accurate description of a multitude of scenarios involving people, vehicles, roads, traffic lights, and so on. This has emerged as the biggest obstacle to the ability of intelligent systems to carry out various complex tasks. The core capabilities of the entire intelligent system, such as information gathering and intelligent cognition, have advanced in recent years thanks to the rapid development of multi-mode sensors [1]. Among them, visible and infrared images, which serve as the primary visual data sources for intelligent systems, are crucial for a variety of perception tasks.

Infrared and visible images have very different imaging principles and feature representations. While visible images can more effectively present the scene texture details and retain the illumination intensity information, infrared images aim to highlight the overall contour characteristics of the object. However, due to hardware and environment factors, blur and halos may appear in infrared images. Therefore, it is crucial to understand how to fully exploit their benefits and combine infrared and visible images.

Additionally, the demands for multimodal fusion and subsequent downstream tasks, such as object detection and semantic segmentation, are growing rapidly with the vigorous development of video surveillance and automated driving; the majority of currently used approaches focus on creating networks or models to improve the visual impact of the fused image, but they ignore the fact that the fused image only matches the human vision, making it hard to meet the perceptual requirements of subsequent tasks, such as object detection and segmentation.

The approach suggested in this paper aims to address the existing practical issues. In order to effectively address the underlying visual issues in challenging environments, this method employs two techniques: multi-layer semantic information guidance and a neural network architecture search. These techniques work together to enhance the visual quality and effects of fused images as well as the performance of downstream tasks.

Aiming to fully utilize semantic information and conduct target detection-oriented fusion research, this paper employs a neural network search scheme and attention mechanism to process various downstream tasks. The main research work and contribution of this paper can be divided into two main parts:

- In order to reduce feature redundancy and preserve complementary information, we designed a multi-level adaptive attention block (MAAB) in the network, which allows our network learning to retain rich features at different scales, and more efficiently and effectively integrate high-level semantic information.
- To discard the limitations of the existing manually constructed neural network structure, we introduce a neural architecture search (NAS) in the construction of the overall network structure, so as to adaptively search the network structure that is suitable for the current fusion task.

This paper will be divided into the following sections.

Section 1 introduces the research background and the significance of IVIF, as well as the subsequent target detection. This section briefly introduces the research status of a deep learning scheme of multimodal image fusion [2,3,4], and puts forward the main work and innovation of this paper.

Section 2 introduces the research status of infrared and visible image fusion, including traditional methods and deep learning methods [5,6,7,8].

In Section 3, an infrared visible image fusion and detection algorithm guided by semantic information is proposed. This section introduces the specific network structure, the construction of loss function, and the design of the search space.

In Section 4, experiments are carried out to verify the effectiveness of the method. Moreover, the final section summarizes this paper.

## 2. Related Works

In this section, we review the current state of research on visible and infrared image fusion methods, specifically divided into two parts: traditional fusion methods and deep learning fusion methods. Moreover, we introduce the work on a neural architecture search.

### 2.1. Fusion Methods Based on a Traditional Approach

IVIF algorithms based on traditional approaches are divided into five categories: the fusion method based on multi-scale transform, sparse representation, subspace, saliency, and other traditional theories.

#### 2.1.1. Fusion Methods Based on Multi-Scale Transform

Due to their unique information extraction tactics, multi-scale transform approaches have had a lot of success in the image fusion field. The multiscale transform acquires the multiscale representation of the input image, then constructs the fused multiscale coefficient using fusion rules. Finally, to acquire the fusion result, the coefficient is inversely converted. Although multi-scale transformation is a well-known and effective technique, it has issues with halo and ghosting when reconstructing images.

Several researchers are altering the multi-scale transformation method to address these issues. To accomplish greater noise reduction and more efficiently extract rich information, Nencini et al. [9] used the curvelet transform to divide source images into multiple wavelet domains. In addition, Ma et al. [10] utilized Gaussian and rolling guidance filters to decompose source images into four scales based on the base layer and detail layer. Then, they employed an advanced visual saliency map to process the base layers and added the detail layers into the base layers using weighted least square optimization. Their algorithm generated fused images that contain more natural details and are more consistent with human visual perception.

#### 2.1.2. Fusion Methods Based on Sparse Representation

Sparse representation methods utilize an over-complete dictionary, which is trained on a large set of high-quality natural images, to sparsely represent the input images. This is in contrast to multi-scale-based fusion methods. This compact formulation is more practical for infrared and visible image fusion processing since it only requires a small number of atoms from the dictionary to describe the image’s information.

Creating an appropriate over-complete lexicon is a highly researched area. Liang et al. [11] provided a solution for various image fusion problems by building a tensor and using singular value decomposition (SVD) to deconstruct the images. A method for learning dictionaries that combines sparse regularization words and low-rank representation was published by Li et al. [12]. Additionally, this method effectively reduces noise interference in findings from medical image fusion and even enhances soft tissue details. Zhu et al. [13] utilized orthogonal matching pursuit, which is achieved by breaking down the multimodal image source to obtain sparse coding of texture components. Liu et al. [14] integrated convolutional sparse representation (CSR) into image fusion to preserve rich details and reduce the impact of misregistration.

#### 2.1.3. Fusion Methods Based on Subspace

A subspace is a region of space whose dimensions are less than or equal to the total dimensions of the space. High-dimensional input image dimensions are reduced by the subspace-based fusion technique so that they can be projected onto a low-dimensional subspace. In order to fuse infrared and visible light, it is necessary to remove some redundant information from the source images. By utilizing the dimensionality reduction representation approach, it is possible to capture the valuable internal structure of the original image while minimizing the execution time and memory costs.

Li et al. [15] used principal component analysis (PCA) to combine low-frequency images that had undergone a morphological transformation, extracting and storing each energy component separately to preserve the brightness information from the source image. Ibrahim et al. [16] used the robust principle component analysis algorithm (RPCA) to split the source image matrix into low-rank and sparse components, producing high and low frequency subband coefficients after fusion in order to reduce some of the noise.

#### 2.1.4. Fusion Methods Based on Saliency

Saliency detection is an intelligent algorithm that simulates the perceptual features of the human visual system and extracts the key elements of human perception from a picture. This bottom-up attention process is generated by the pixel and its neighbors, and researchers aim to preserve the target’s position in the salient zone.

Saliency can inevitably be used to extract a considerable target region from a source image. For instance, Liu et al. [17] used a sparse representation framework to fuse images using global and local multi-scale saliency monitoring while simultaneously applying the saliency model to the source images of two modes. Shibata et al. [18] used a super-pixel based saliency model to extract crucial areas from an infrared image while keeping the general information of targets.

Furthermore, a fusion method uses importance to calculate the weight. In this process, the source image is first multi-scale transformed into the overall target layer and the detail information layer. Afterward, the saliency map is extracted using the saliency model, and transformed into the weight map to create the fused two layers; finally, the fused image is recreated using multi-scale inverse transformation.

Gan et al. [19], for example, employed phase consistency as a method of saliency detection to produce the saliency layer of the whole layer and the detail and then used the guided filtering on the saliency layer to obtain the fusion weighted map for fusion.

#### 2.1.5. Fusion Methods Based on Other Traditional Theories

In addition to the fusion processes mentioned above, many other fusion methods exist that offer novel concepts for infrared and visible light fusion. Researchers have presented fuzzy theory and practice as a means of addressing the challenge of image blurring caused by the fusion of high-quality source images. For example, Rajkumar et al. [20] proposed a fusion method based on non-sampled contour transformation and fuzzy logic, along with an adaptive average weighted fuzzy rule to guide the fusion process.

The amount of information conveyed by entropy is an indicator of its richness. It can be used to evaluate the extent to which data from the source image is transmitted to the fused image. Zhao et al. [21] developed a cost function that utilizes the global maximum entropy as the primary term and gradient constraint as the regularization term, to maximize the transmission of information from the source image to the fused image.

The primary concept of morphology is to extract the form or feature in an input image for subsequent operation and processing using unique structural components. Bai et al. [22] suggested a morphological center-based fusion approach. To produce the final fusion features for fusion, the method first employs the morphological center operator to detect the bright and fuzzy characteristics of the source image, and then extracts multi-scale features using the correlation coefficient technique.

### 2.2. Fusion Methods Based on Deep Learning

The development of deep learning has led to outstanding achievements being made in many fields of computer vision [23,24,25,26,27,28]. The IVIF algorithms based on deep learning are divided into four categories: the fusion method based on a pretrained deep neural network, autoencoder, end-to-end model, and the generative adversarial network.

#### 2.2.1. Fusion Methods Based on Pretrained Deep Neural Network

This kind of method transforms the image feature extraction operation from the manually designed mathematical operations to the pre-trained deep neural network. It uses the multi-channel feature map generated by the neural network to achieve rich information representation, and reconstructs the feature map through the fusion strategy to generate results. The key to this kind of method lies in the design of the neural networks and fusion rules.

In the image classification task, there are very mature, large-scale visible light databases with multiple scenes and targets, and they have been used to train neural networks for classification. Common ones include the linear multilevel VGG convolutional neural network [29], ResNet based on the residual skip connection [30], DenseNet based on a dense link [31], etc. Researchers working on image fusion have also started using pre-trained feature extraction networks as methods for extracting features in the fusion framework. For example, Li et al. [32] proposed a fusion framework based on the zero-phase component analysis using the pre-trained ResNet-50 network as the feature extraction network. They also apply the VGG network for multi-level feature extraction [33].

However, such methods have obvious limitations. First, the pre-trained network parameters limit the infrared and visible light fusion since these networks are trained for image classification. Secondly, the deep neural network suffers from the degradation phenomenon. When using its deep-seated features for fusion, it will inevitably be disturbed by the lost and redundant/useless features. How to reduce this impact in applications also needs special consideration [34,35,36].

#### 2.2.2. Fusion Methods Based on Autoencoder

In the network structure based on the autoencoder, the main parts are the encoder and the decoder. The overall process is that the source image pair is input into the encoder, transformed into a feature map representation, and is fused at the feature level through the set fusion strategy; the fused feature map is then reconstructed by the decoder to generate the fusion result map.

The training strategy for the self-coding network is flexible. Encoders and decoders with different network structures are designed and trained on large-scale datasets to obtain network parameters with good generalization performance. After the training, in the test phase, the learned parameters of the network are fixed, and the fusion strategy is added between the encoder and the decoder to perform the fusion operation.

Compared with the fusion algorithm of the pre-training network, the encoder and decoder have good scalability. Li et al. [37] took the lead in using self-coding networks and dense convolution blocks for feature-level fusion. The use of dense blocks enriches the feature image information. Zhao et al. [38] proposed a fusion network based on the self-encoder. The encoder decomposes the image into the background and details feature images that contain low-frequency and high-frequency data, respectively, restoring the image through the decoder after fusion.

This method also has some disadvantages. First, the manually set fusion strategy cannot reduce the difficulty and time cost of the algorithm in the operation. Second, this method fails to address the challenge posed by the lack of multimodal images in the training data, which ultimately limits the network’s ability to adapt to a wider range of parameters.

#### 2.2.3. Fusion Methods Based on the End-to-End Model

The end-to-end model attempts to integrate the multi-step model into a single model to directly realize feature extraction, fusion, and image reconstruction in the algorithm. Li et al. [39] improved the self-coding fusion algorithm framework to an end-to-end fusion framework. They replaced the artificially designed fusion part of the original network with a learnable fusion network and trained it separately using the self-coding algorithm training method. This method provides a new approach to improve the disassembly of the end-to-end model. Zhang et al. [40] adopted the end-to-end fusion method in the multimodal image fusion framework, using double-layer convolution for feature extraction and deciding which fusion method to use based on the image mode.

Although the end-to-end model is widely used, it also has some issues. Compared to the step-by-step multi-module model, the specific process in the end-to-end model has poor interpretability, making it difficult to determine the contribution of each part to the fusion result.

#### 2.2.4. Fusion Methods Based on the Generative Adversarial Network

The generation adversarial network (GAN) [41] is composed of a generator and a discriminator. The generator aims to generate pictures that are close to the real value, and the discriminator aims to judge the false–true value output by the generator. The two play games with each other to restrict each other and learn together.

Ma et al. [42] introduced this concept into infrared and visible light fusion. Considering that this is an unsupervised task without truth value, they combined the detailed information of visible light with the overall infrared information to jointly create a composite loss function to constrain the generation of a countermeasure network.

Just as the encoders in the self-encoder structure can design their own encoders for different modes of source images to better extract features, Ma et al. [43] split the discriminator in the generated countermeasure network into the detailed discriminator and overall discriminator, which further restricted the generator from generating a fused image, with overall perceptions tending to the range of infrared thermal targets and local details tending to the rich texture of visible light.

Liu et al. [44] designed a double discriminator network and a new double-layer optimization mathematical model for downstream target detection based on infrared and visible light fusion at the level of decision-making fusion. Their two-level optimization model and cooperative training design made their method perform better in subsequent target detection tasks.

On the premise of good training, this type of method can fit the distribution of training samples well and generate more realistic fusion images. Additionally, its loss function design is relatively simple. However, its disadvantage is that the training process can be unstable, and the training data samples at this stage may be limited and not diverse enough.

### 2.3. Neural Architecture Search

Lee et al. [45] introduced a differentiable neural architecture search method, which optimizes hyperparameters in the search space through gradient descent, significantly reducing the computational complexity and time cost of neural architecture search. This method has applications in various fields, including image classification, object detection, and speech recognition. Liang et al. [46] presented ProxylessNAS, a new differentiable neural architecture search method that does not require a predefined search space and can be optimized end-to-end for target tasks and hardware. It is mainly used for image classification tasks and can quickly obtain efficient neural network architectures.

## 3. The Proposed Method

### 3.1. Method Motivation

As mentioned in the introduction, the goal of IVIF is to retain complementary information and remove redundant information from the two modal images. At present, most fusion methods achieve high-quality fusion results by increasing the network depth or manually designing various loss functions. We anticipate that the foreground object in the fused image will be nearer to the infrared image, consistent with the top-down attention mechanism of human vision, which directs our focus toward the highlighted regions of the infrared image. In addition, saliency representation has achieved great success in the field of computer vision. It extracts salient features in images by similar means. For infrared and visible image pairs, the internal specific contrast can distinguish between the foreground target and background details using significance representation.

Moreover, it is well known that high-level semantic information can effectively guide subsequent downstream tasks. When performing infrared-visible image fusion and subsequent target detection simultaneously, incorporating such rich semantic information is expected to enhance feature representation and improve target detection results.

Finally, considering that manually designed network structures may have difficulty adapting to the introduction of high-level semantic information, we propose using neural architecture search with a high upper limit and good effectiveness to determine sub-operations. Thus, we propose a multi-level attention-guided search fusion network based on semantic information. By incorporating saliency information constraints into the loss function, designing multi-level attention modules, and introducing NAS, we expect that this method can generate visual fusion results with clear thermal targets and rich real details.

### 3.2. Network Architecture

**Figure 1:**
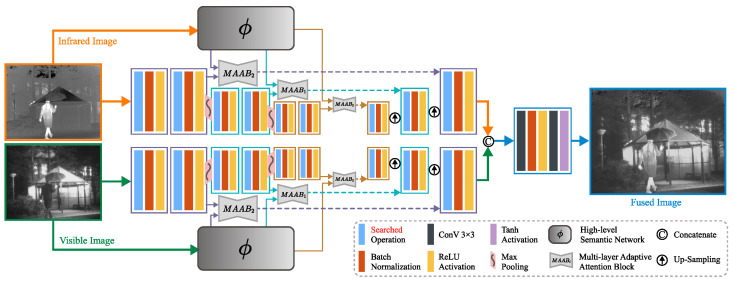
The overall architecture of the proposed method.

The network structure is composed of three parts: a high-level semantic network, a multi-layer adaptive attention block, and a searched residual network. The overall structure of the whole network is shown in Figure 1; each part will be introduced separately below.

#### 3.2.1. High-Level Semantic Network

In this paper, we use the pre-trained VGG16 network as the basic framework of the high-level semantic network ϕ. VGGNet has a simple structure, which is suitable for image classification tasks. However, in other research fields of computer vision, researchers found that networks with excellent pre-trained weights have outstanding generalization performances when migrating to other image data. Therefore, VGGNet is still often used to extract high-level image features.

**Figure 2:**
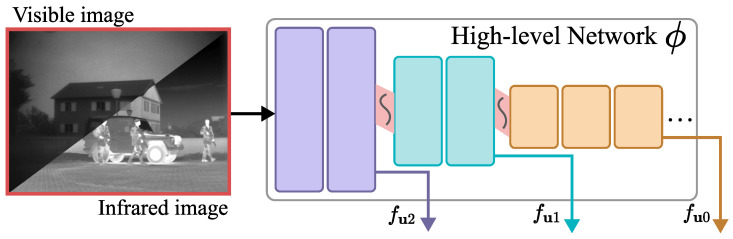
The overall architecture of the high-level semantic network(part of VGG16).

As shown in Figure 2, we extract high-level semantic information at three different scales in the VGG16. The larger the size of the feature map, the richer the details and textures of the image, while the smaller the size of the feature map, to some extent, can reflect the overall pixel distribution and significant areas. The extracted features are $f_{u2}$, $f_{u1}$,$f_{u0}$, and their dimensions are (64, H, W), (128, H/2, W/2), and (256, H/4, W/4), respectively.

#### 3.2.2. Multi-Layer Adaptive Attention Block

To adapt the image features of the two modes to their semantic information at different scales, this paper proposes a multi-layer adaptive attention block (MAAB) [47,48].

**Figure 3:**
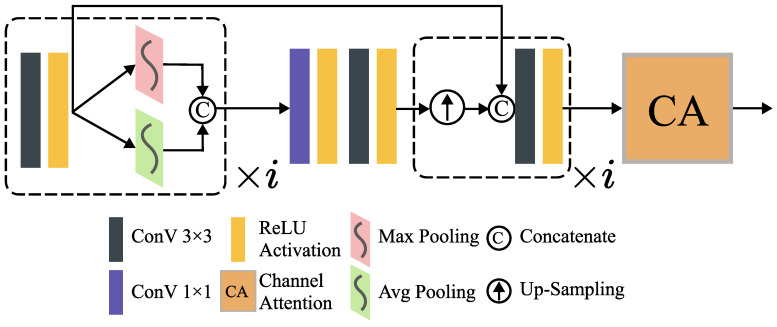
The architecture of $MAAB_{i}$. i=2,1,0.

As shown in Figure 3, under the guidance of a similar spatial attention mechanism, the module is designed to have an adaptive structure for processing at different scales, where *i* denotes the number of downsampling operations applied. As the feature map size continuously decreases, we reduce it to the order of i=2,1,0 to ensure a minimum feature size and prevent excessive loss of structural information.

Specifically, the module combines the feature information of different modes through the searched convolution layer and then obtains the information by using the average pool feature and the maximum pool feature. After downsampling operations for *i* times, the module can fully contain local and global feature information. Jump links are added at the corresponding convolution layer to enrich the amount of information and make up for some losses. Then these intermediate features, containing spatially significant information, are sent to the channel attention module introduced below to obtain richer and more effective deep-seated semantic information [34,35,36].

**Figure 4:**
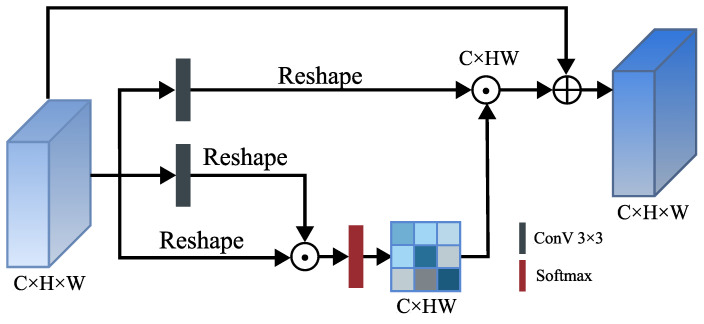
The architecture of channel attention.

As shown in Figure 4, the input features undergo a squeeze operation on the channel domain, generating a channel descriptor by integrating the feature map in the spatial dimension. This descriptor embeds the global distribution of channel characteristic response, allowing the network to use information from the global receptive field. The two-channel descriptors are multiplied element-wise and passed through a softmax function to generate a weight graph representing the degree of dependence between channels through the self-gating mechanism based on the dependence between channels. The weight graph is multiplied by another channel descriptor point and reshaped into the original three-dimensional feature representation. Similarly, in order to reduce information loss in the process, there is a jump link between the original input characteristic map and the final output result [49].

#### 3.2.3. Searched Residual Network

The method employed in this study uses different scales of semantic information, and determining how to use this information manually can be challenging. To improve the network’s adaptability to semantic information, the study incorporates a neural network structure search to determine the network structure adaptively [47,48].

Considering that the original task of the directed acyclic graph used by darts is image classification, and residual blocks and dense link blocks have exhibited outstanding performance in image fusion tasks, the search network proposed in this paper fixes the form of the residual network, and only searches for the weight of the single-layer convolution. This cannot only reduce the search time and operation cost but also better adapt NAS to the task of image fusion.

**Figure 5:**
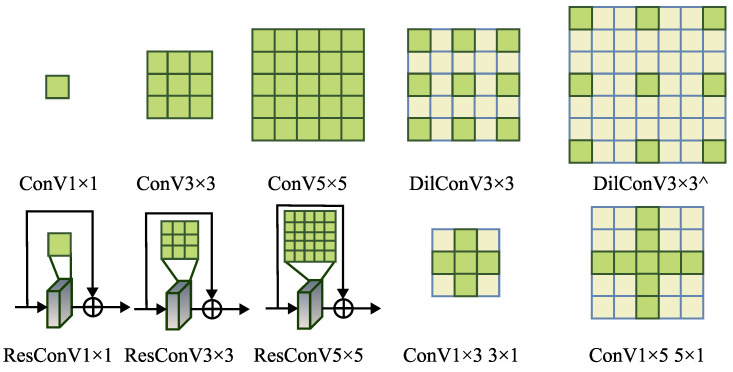
The channel attention architecture.

Constructing an effective search space is a critical task in the neural architecture search (NAS) domain. A well-designed search space should balance the complexity of the candidate operations with their ability to improve the network architecture. In this paper, we present ten effective and efficient operations that were selected from the search space, as illustrated in Figure 5:

- ConV 1 × 1
- ConV 3 × 3
- ConV 5 × 5
- DilConV 3 × 3
- DilConV 5 × 5
- ResConV 1 × 1
- ResConV 3 × 3
- ResConV 3 × 3
- ConV 1 × 3 3 × 1
- ConV 1 × 5 5 × 1

We integrated these operations, referred to as searched operations, into the encoders and decoders of both visible and infrared networks, as demonstrated in Figure 1.

The process of discovering searched operations involves three steps. First, a mixed operation is constructed by computing a weighted sum of the ten operations in the search space, with the weight coefficients represented by a weight matrix. Second, the training loss, which incorporates the weight matrix, is minimized through gradient descent optimization. The weight matrix is updated iteratively until convergence. Finally, the operation with the maximum weight in the weight matrix is chosen as the Searched Operation, and the search process is terminated.

Once we have identified each searched operation through the search process, the architecture of the visible and infrared networks is fully determined. We then retrain the network using the training data to obtain the training results. Integrating the searched operations leads to better performance compared to using manually designed convolutional operations in the encoder and decoder. The experimental results demonstrate the effectiveness and efficiency of this NAS approach.

### 3.3. Loss Function

The setting of the loss function is an important part of solving computer vision problems by using the deep learning method. This section will introduce each part of the loss function. In this section, I represents the image, and $I_{A}$, $I_{B}$, and $I_{F}$ represent the input visible image, infrared image, and fused image, respectively.

#### 3.3.1. Pixel Loss Function

Considering that this is an image fusion task, we use the most common pixel-level constraint to guide the network training, which is defined as follows:

(1) $$L_{pixel}=\frac{1}{HW}{\left|\left|I_{F}-I_{A}\right|\right|}_{2}^{2}+\frac{1}{HW}{\left|\left|I_{F}-I_{B}\right|\right|}_{2}^{2}$$

#### 3.3.2. Structure Loss Function

Overall, we expect the final fusion image to be structurally similar to the source image, because the most commonly used structural similarity index is used as the loss function, which is defined as follows:

(2) $${SSIM}_{F,X}=\sum_{x,f}\frac{2\mu_{x}\mu_{f}+C_{1}}{\mu_{x}^{2}+\mu_{f}^{2}+C_{1}}\cdot \frac{2\sigma_{x}\sigma_{f}+C_{2}}{{\sigma_{x}}^{2}+{\sigma_{y}}^{2}+C_{2}}\cdot \frac{\sigma_{xf}+C_{3}}{\sigma_{x}\sigma_{f}+C_{3}},$$

(3) $$L_{SSIM}=\left(1-SSIM_{F,A}\right)+\left(1-SSIM_{F,B}\right).$$

${SSIM}_{F,X}$ represents the structural similarity between the source image X (X represents the source images A and B) and the fused image F, and *x* and *f*, respectively, represent the image patches of the source image and the fused image in the sliding window, $\sigma_{x,f}$ is the covariance of the source image patch and the fused image patch, $\sigma_{x}$ and $\sigma_{f}$ represent the standard deviations of plaque, $\mu_{x}$ and $\mu_{f}$ are the average values of the source image patches and fused image patches. $C_{1},C_{2},C_{3}$ are the parameters used to stabilize the algorithm [50].

The brightness information of an object’s surface is related to the illumination of its environment and the object’s reflection coefficient. In natural scenes, an object’s structure and material are generally independent of ambient illumination. This means that the reflection coefficient is only related to the object itself. By taking this into consideration, we can explore the structural information in an image by separating the influence of illumination on the object.

From an image composition standpoint, the structural similarity index (SSIM) characterizes structural information as a feature that portrays the object’s arrangement in the scene, while being invariant to brightness and contrast. It models image distortion by combining three distinct elements: brightness, contrast, and structure. The mean is used as the estimate of brightness, the standard deviation as the estimate of contrast, and the covariance as the measure of structural similarity. Compared with the traditional measurement indicators, such as PSNR, the structural similarity is more consistent with the judgment of human eyes on image quality in the measurement of image quality.

#### 3.3.3. Gradient Loss Function

In addition, image gradient information can be used to represent texture details and scene structures. In order to enrich the details in the fused image, this paper uses gradient loss [51] to constrain texture factors, as follows

(4) $$L_{grad}=\frac{1}{HW}{\left|\left|\nabla \;I_{F}-Max(|\nabla \;I_{A}|,|\nabla \;I_{B}\left|\right)\right|\right|}_{2}^{2},$$

where ∇ is defined as the gradient operation using the Sobel operator. The Sobel operator is an important processing method in the field of computer vision, which is mainly used to obtain the one-step degree of the digital image. HW represents the product of the height and width of the image.

Technically, it is a discrete difference operator used to calculate the approximate value of the gradient of the image brightness function. It weights the gray values of each pixel in the image, and the extreme value obtained is the edge for edge detection. Applying this operator at any point in the image will produce the corresponding gradient vector. In general, the Sobel operator produces good detection results and has a smoothing effect on noise, which is why it is widely used in image fusion. This paper calculates the Sobel operator in the X direction and Y direction, respectively, and combines them to obtain the final result. As shown above, in the background, the same part often has large gradient differences in images of different modes. We take the maximum value of the gradient information of the two modes to calculate the loss function, which can reduce the impact caused by modal differences.

#### 3.3.4. Target-Aware Loss Function

In this paper, we propose a weighted average technique based on a visual saliency map (VSM) to design the pixel-level loss function. VSM [52] can depict and highlight visual structures, regions, or objects in images, making it useful in various computer vision and computer graphics applications. In image fusion, VSM can reflect the salient features of the image, providing a regional semantic information constraint based on the distribution of pixel features.

Considering the simplicity and effectiveness of the method, this paper uses this method [53] to construct VSM. The algorithm defines pixel-level saliency based on the contrast of pixels with all other pixels. Let $P_{i}$ represent the intensity value of a pixel *i* in image *I*. The salient value V(i) of pixel *i* is defined as

(5) $$V\left(i\right)=\left|P_{i}-P_{1}\right|+\left|P_{i}-P_{2}\right|+\cdots +\left|P_{i}-P_{N}\right|,$$

where *N* denotes the total number of pixels in image *I*. The saliency values of two pixels are equal if two pixels have the same intensity value. Thus, we can rewrite this as follows:

(6) $$V\left(i\right)=\sum_{j=0}^{G-1}M_{j}\left|P_{i}-P_{j}\right|,$$

where *j* denotes the pixel intensity, $M_{j}$ represents the number of pixels whose intensities are equal to *j*, and G is the number of gray levels (256 in this paper). Then, V(i) is normalized to [0, 1]. Let $V_{B}$ denote the VSMs of the infrared images. We can denote the target-aware loss function as follows:

(7) $$L_{target}=\frac{1}{HW}{\left|\left|I_{F}\cdot V_{B}-I_{B}\cdot V_{B}\right|\right|}_{1}$$

#### 3.3.5. Total Fusion Loss Function

Consequently, combining all of the loss functions above, the following total fusion loss function guides the learning of image fusion.

(8) $$L_{fusion}=L_{pixel}+\alpha L_{SSIM}+\beta L_{grad}+\gamma L_{target},$$

where $L_{pixel}$ is the pixel loss, and $L_{SSIM}$ and $L_{grad}$ are the structure loss and gradient loss, respectively. α, β, and γ are the trade-off parameters. In the relevant settings of the neural network search, we also use this loss function as the loss function of the training set and the val set. The definitions are as follows:

(9) $$L_{train}=L_{fusion}=L_{val}$$

In this way, the overall goal of the network can be consistently ensured, and the convergence of the structure and parameters can be facilitated.

## 4. Experiments

We conducted experimental evaluations on three datasets, namely TNO, Roadscene, and M3FD. For the image fusion task, we selected 180 images from the 4500 images in the 3 datasets and converted them to grayscale. To enhance the data content and make better use of the images, we randomly cropped these images to generate 20 K pictures of size 64 × 64 for network training. During training, these image blocks were normalized to [−1, 1] before being fed to the network.

In the search process of the neural architecture search, we used Adam [54] as the optimizer on the training set and set the learning rate as 1 × 10${}^{-5}$. In the validation set, we used SDG as the optimizer and set the learning rate and the weight attenuation of the weight matrix as 1 × 10${}^{-2}$ and 1 × 10${}^{-4}$, respectively. The batch size during training was set to 16, and the number of epochs was set to 100. After determining the structure, we used the Adam optimizer during training and set the learning rate to 2 × 10${}^{-5}$. The batch size during this training was set to 64, and the number of epochs was set to 20.

In object detection, we used 4200 pairs of images labeled in M3FD for network training. We used YOLOv5 as the object detection network and we used the pre-trained YOLOv5s model. In object detection training, the image size was set to 320 × 320, and other parameters were carried out according to the original data provided by the official.

Our approach was implemented on PyTorch with an NVIDIA Tesla V100 GPU. The tuning parameters α, β, and γ were set to 0.3, 0.5, and 1.2, respectively.

**Figure 6:**
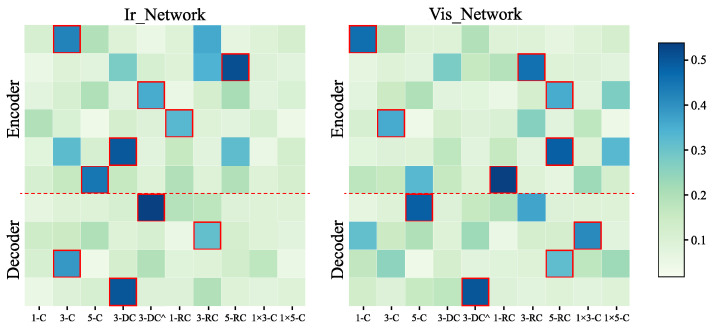
Heat map of search results by the training strategy.

First, as shown in Figure 6 below, we exhibit a heat map of the neural network search results. The heat map depicts the weight matrix, which was introduced in Section 3.2.3. The left subfigure illustrates the infrared network, while the right subfigure portrays the visible network. Each column in the figure corresponds to ten operations in the search space, and each row corresponds to a searched operation in either the encoder or decoder. The intensity of the color used in the figure signifies the weight of the operation, with darker shades denoting greater weights. Ultimately, the operation with the highest weight is selected as the searched operation, which is highlighted by the red box.

### 4.1. Results of Infrared and Visible Image Fusion

We evaluated the fusion performance of our method by comparing it with seven state-of-the-art methods: DenseFuse [37], FusionGAN [42], RFN [39], GANMcC [55], IFCNN [40], MFEIF [56], and U2Fusion [57].

#### 4.1.1. Qualitative Comparisons

(1) Qualitative Comparisons on TNO Datasets

**Figure 7:**
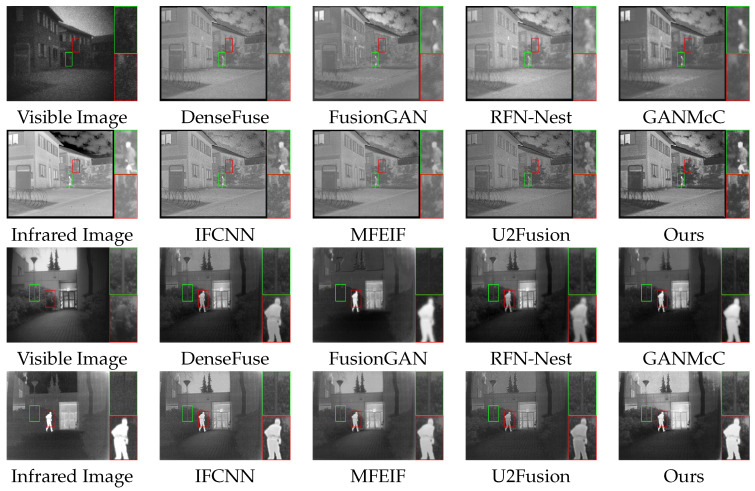
Qualitative comparison of typical image pairs in the TNO dataset.

The intuitive qualitative results on two typical image pairs from the TNO dataset are shown in Figure 7. The boxes in the figure indicate the target objects and textured background in the fused image that are of our interest.

The first pair of famous images depicts a residential area under night surveillance. The infrared image clearly shows useful information that is difficult to distinguish in visible images, such as human figures, clouds, shrubs, and wire circles. As shown in the red boxes, our method effectively preserves the contrasted information of the image, and the fused image indicates the shrubs as background with an even distribution of pixels, while other methods, such as FusionGAN and GANMcC, generate blurred backgrounds. As shown in the green boxes, our approach outlines target objects that are more apparent, while other methods, such as RFN-Nest, are greatly affected by the visible images, and the outline of the target object is blurred and difficult to identify.

The second pair of images depicts a daytime surveillance scene. The human figure in the visible image blends into the background, while the target object is evident in the infrared image. The outline of the street light pole in the infrared image is difficult to identify, and the outline is more apparent in the visible image. As shown in the red box, our method sharpened the contours of the target object compared to the visible image and effectively absorbed the valuable information from the infrared light image, while other methods, such as RFN-Nest and U2Fusion, generated blurred low-brightness target objects. As shown in the green box, our method effectively preserves the gradient information and clearly shows the outline of the street light pole as a detailed background, while other methods, such as FusionGAN, generate fused images with uneven distribution of pixels in the background, making it difficult to distinguish the outlined details.

(2) Qualitative Comparisons on Roadscene Datasets

**Figure 8:**
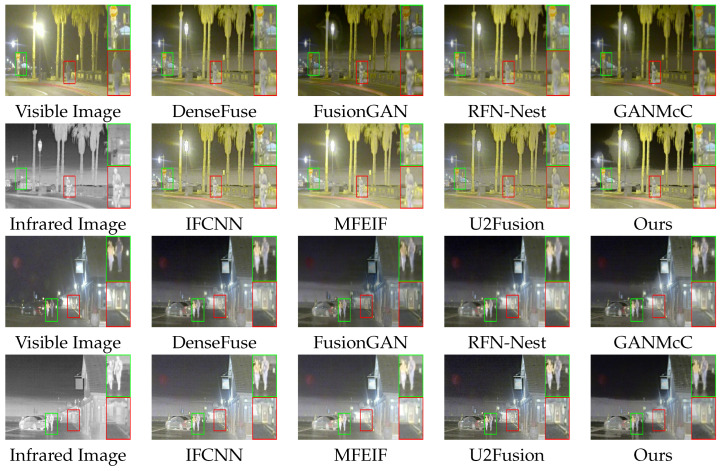
Qualitative comparison of typical image pairs in the Roadscene dataset.

The intuitive qualitative results of two typical image pairs from the Roadscene dataset are shown in Figure 8. The boxes in the figure indicate the target objects and textured background in the fused image that are of our interest. The grayscale image, as the fusion result, is treated as the Y channel and merged with the Cb and Cr channels of the visible light image to form a YCbCr image, which is then converted to the RGB channel to obtain the color fusion image.

Two classic pairs of images depict street scenes at night. In contrast to the previous pairs, both pairs include artificial light sources, setting a unique barrier to image fusion.

In the first group of images, the target object in the visible image is not identifiable, while the background details in the infrared image have a chaotic pixel distribution. In the second group of images, the vehicle in the visible image blends in with the blackness, while the overexposure caused by the artificial light source blurs the background details in the infrared images. Our method shows stronger contrast and richer color details compared to FusionGAN and GANMcC, as shown in the green box in the first set and the red box in the second set. It also avoids overexposure that causes ghosting compared to MFEIF. Moreover, as shown in the red box in the first group and the green box in the second group, our method effectively enhances the contours of the target object.

(3) Qualitative Comparisons of M3FD Datasets

**Figure 9:**
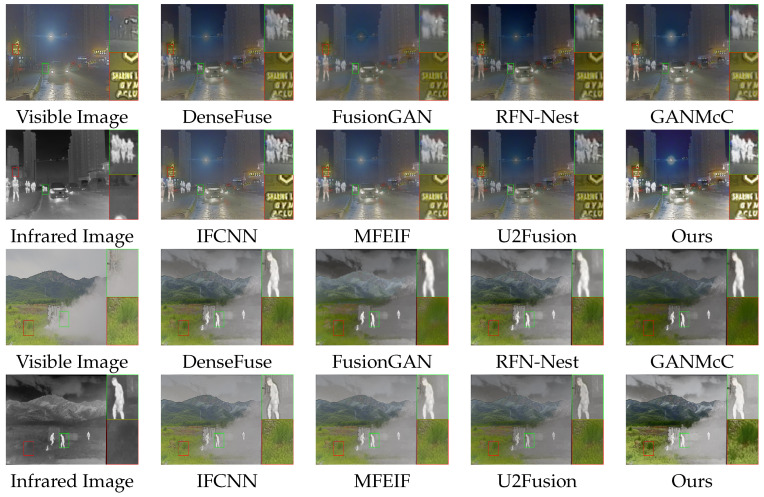
Qualitative comparison of typical image pairs in the M3FD dataset.

The intuitive qualitative results of two typical image pairs from the M3FD dataset are shown in Figure 9. The boxes in the figure indicate the target objects and textured background in the fused image that are of our interest.

These two sets of images evaluate the effectiveness of different fusion approaches under typical and unique barriers.

The first set of images shows a night street scene. The visible image has overexposure due to artificial light sources, making it difficult to recognize the human silhouette. The infrared image cannot capture the artificial light sources and loses text information on the signboard. As shown in the green box, our method generates the fused image with the brightest and most visible target object compared to other methods. As shown in the red box, our method produces an image with the highest contrast, good distribution of background details, and precise text information on the signboard.

The second set of images depicts a mountain scene during the daytime. The presence of smoke in the visible image makes it impossible to identify the target object. Still, the infrared image can capture the target object information through the smoke. As shown by the green box, our method fully absorbs the knowledge of the target object in the infrared image, with clear contour edge information and good brightness. As shown by the red box, the fused image generated by our method has high contrast, good pixel distribution, and rich color details compared to other methods in representing background details.

#### 4.1.2. Quantitative Comparisons

In order to make a more comprehensive quantitative comparison, we selected six common fusion indicators in the field of image fusion as the evaluation benchmark, including three referenced indexes: mutual information (MI) [58], structural similarity (SSIM) [50], and the sum of correlation differences (SCD) [59], and three non-referenced evaluation indexes: entropy (EN) [60], standard deviation (SD) [61], and spatial frequency (SF) [62].

- The quantity of information transmitted from the source image to the fused image is measured using MI. A larger MI indicates that more information from the source image pair is maintained in the fused image.
- The human visual system is sensitive to picture loss and distortion, and this is modeled using SSIM. It has a good correlation with fusion performance.
- SCD displays the correlation between the source and fused images. A larger SCD indicates a higher fusion performance.
- EN measures the information in the fused image. A higher EN typically denotes improved fusion performance.
- The contrast and pixel distribution of the fused image are reflected by SD. A larger SD typically denotes a more aesthetically pleasing fused image.
- SF represents the overall gradient distribution of the image in the spatial domain. The texture and edges become richer as the SF becomes larger.

The specific results are shown in Table 1 and Table 2 below.

In the TNO dataset, as shown in Table 1, our method consistently delivers the highest or second-highest mean compared to the other methods. The lower standard deviation, on the other hand, illustrates the consistency of our approach across cases. Our method specifically obtains the highest EN, SD, and SF, which shows that it produces fused images with rich information, good pixel distribution, strong contrast, and rich texture. SSIM reflects the pleasing visual effect of our fusion method.

In the Roadscene dataset, as shown in Table 2, our approach yields the highest average values under MI, EN, SD, and SF evaluations. The highest MI and EN values show how effectively our method can transfer information from the source images to the fused images. The highest SD and SF values demonstrate how the fused images generated by our method are aesthetically pleasing and maintain their sharp edges and rich textures.

### 4.2. Results of IVIF and Object Detection

In the ensuing target detection section, we conducted the following actions to ensure that the experiment was fair: 300 epochs were retrained based on the pre-trained YOLOv5s model for each fusion method, and their corresponding detection network parameters were obtained before detection. That is, a pre-trained YOLOv5s model was used and fine-tuned on the fused images.

In the dataset used in this paper, only the M3FD dataset has object annotation, so relevant experiments were carried out on this dataset. Different from our previous work, this time we enabled a new training set and test set. The 4200 images in the dataset are divided into a test set and a training set according to the 800/3400 images and scenes. Each scene in the test set has not appeared in the training set.

The quantitative results of target detection in the M3FD dataset are shown in Table 3. Almost all fusion approaches have shown excellent detection results in general. Except for particular label categories, the detection accuracy of fusion approaches outperformed that of using solely visible or infrared pictures. By highlighting the representation of infrared thermal objects and activating highly rich information in the modes through high-level semantic information, our method has greater advantages in this dataset featuring challenging scenes.

### 4.3. Ablation Studies

#### 4.3.1. Study on Model Architectures

We study our method’s model architecture and further validate the effectiveness of several individual components as shown in Table 4. The introduction of NAS and the MAAB module built for semantic information is the focus of the network structure presented in this study. For these two components, we built appropriate ablation.

**Figure 10:**
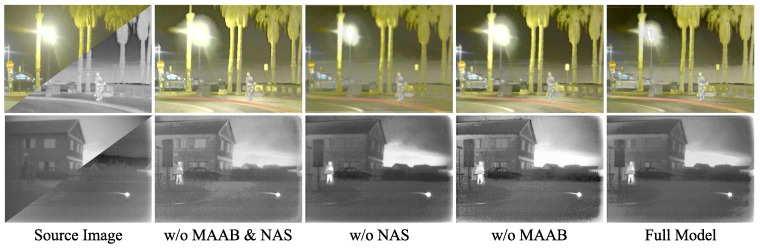
Qualitative comparison of results using different networks.

The initial single-layer search convolution layer is replaced with ConV 3 × 3 (stride = 1) operation when NAS is not used. When MMAB is not used, a combination of ConV 3 × 3 (stride = 1) + BN + ReLU is used instead. Figure 10 shows the visualization results of fused images generated by models with different ablation degrees, where, w/o means “without”.

Without MAAB, the utilization of high-level semantic information has some adaption issues, and the overall contrast and color are poor. Without NAS, it is impossible to make greater use of semantic information with the original features, resulting in a significant loss of detail. These two components have favorable effects on the ultimate fusion result.

The relevance of MAAB and NAS in the network can also be seen in the quantitative comparison presented in the table. Our overall model’s final findings came in second place. The M1 model creates too much noisy pixel distributions, which interferes with the findings, thus ’en’ is not the greatest value.

#### 4.3.2. Analyzing the Training Loss Functions

**Figure 11:**
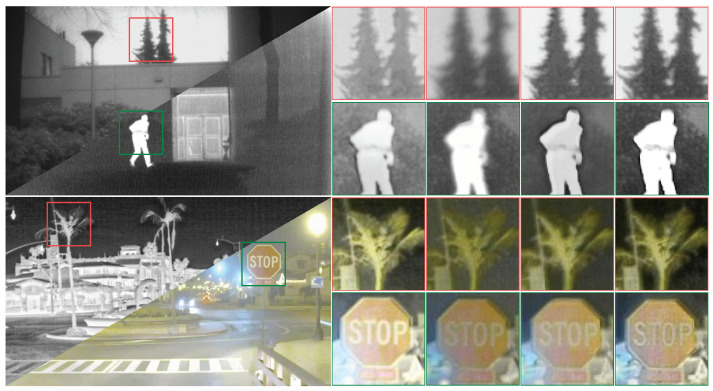
Qualitative comparison of loss functions. From left to right: w/o $L_{SSIM}$, w/o $L_{grad}$, w/o $L_{Target}$ and the $L_{fusion}$.

We discuss the impact of different loss functions on our method. The resulting image is substantially deteriorated without SSIM limitations, as seen in Figure 11; moreover, the contrast and brightness are drastically altered. The edge of the picture becomes blurred without the gradient limitation, and more information is lost. The brightness of the characters in the image is lowered without the significant target limitation supplied by VSM, as is the contrast with the environment, altering the viewing effect.

Overall, the loss functions we investigated do their jobs and have a significant impact on the training process.

## 5. Discussion

Overall, the paper presents a valuable contribution to the field of IVIF and object detection by proposing a multi-level structure search attention fusion network based on semantic information guidance. Future research could expand on these findings to enhance the accuracy and robustness of object detection algorithms in fused images under challenging conditions.

## 6. Conclusions

In this paper, a multi-level structure-search attention fusion network based on semantic information guidance is proposed. The multi-level adaptive attention block (MAAB) is designed in the network to reduce feature redundancy and preserve complementary information. The neural architecture search (NAS) is introduced in constructing the overall network structure to eliminate the limitations of the existing manually constructed neural network structure. Extensive experiments on the new dataset (M3FD) show that our fusion method has achieved advanced performance in both subjective and objective evaluations, and the mAP in the target detection task is improved by 0.5%.

## Figures and Tables

**Table 1:** Quantitative comparison display of the TNO datasets. The best result is highlighted in red whereas the second-best one is highlighted in blue.

	MI	EN	SD	SF	SSIM	SCD
DenseFuse	0.867 ± 0.28	6.832 ± 0.31	34.419 ± 6.84	9.362 ± 2.86	0.437 ± 0.07	1.404 ± 0.11
FusionGAN	0.393 ± 0.14	6.611 ± 0.33	30.633 ± 7.74	6.313 ± 2.38	0.415 ± 0.06	1.359 ± 0.09
IFCNN	0.548 ± 0.16	7.033 ± 0.27	37.272 ± 5.39	9.325 ± 2.47	0.454 ± 0.10	1.300 ± 0.07
GANMcC	0.782 ± 0.20	6.697 ± 0.34	31.764 ± 6.49	6.301 ± 1.47	0.424 ± 0.03	1.340 ± 0.09
RFN-Nest	0.774 ± 0.21	7.038 ± 0.25	37.438 ± 8.05	5.941 ± 1.83	0.367 ± 0.07	1.441 ± 0.11
U2Fusion	0.793 ± 0.25	7.080 ± 0.19	37.509 ± 6.94	10.366 ± 4.00	0.451 ± 0.05	1.274 ± 0.08
MFEIF	0.957 ± 0.35	6.784 ± 0.49	34.340 ± 10.21	7.673 ± 2.27	0.399 ± 0.09	1.360 ± 0.10
Ours	0.948 ± 0.22	7.140 ± 0.35	42.400 ± 13.09	10.378 ± 3.79	0.457 ± 0.06	1.426 ± 0.08

**Table 2:** Quantitative comparison display of the Roadscene datasets. The best result is highlighted in red whereas the second-best one is highlighted in blue.

	MI	EN	SD	SF	SSIM	SCD
DenseFuse	0.877 ± 0.23	7.271 ± 0.21	45.664 ± 6.44	10.513 ± 3.72	0.497 ± 0.03	1.671 ± 0.07
FusionGAN	0.676 ± 0.13	7.113 ± 0.23	39.790 ± 5.72	8.415 ± 2.98	0.485 ± 0.04	1.641 ± 0.08
IFCNN	0.636 ± 0.14	7.278 ± 0.17	47.414 ± 7.07	10.334 ± 2.75	0.529 ± 0.03	1.579 ± 0.15
GANMcC	0.964 ± 0.34	7.301 ± 0.22	46.872 ± 6.65	8.817 ± 1.82	0.421 ± 0.03	1.367 ± 0.09
RFN-Nest	0.926 ± 0.25	7.307 ± 0.22	48.832 ± 6.50	7.366 ± 2.36	0.502 ± 0.02	1.602 ± 0.03
U2Fusion	0.823 ± 0.25	7.275 ± 0.22	42.876 ± 7.39	13.143 ± 2.92	0.526 ± 0.04	1.726 ± 0.12
MFEIF	0.988 ± 0.27	6.076 ± 0.34	40.263 ± 8.83	8.522 ± 3.03	0.530 ± 0.03	1.590 ± 0.03
Ours	1.08 ± 0.20	7.354 ± 0.30	50.039 ± 4.82	13.679 ± 2.52	0.496 ± 0.03	1.667 ± 0.02

**Table 3:** Quantitative results (precision) of object detection in the M3FD dataset among all of the image fusion methods plus detectors. The best result is highlighted in red whereas the second-best one is highlighted in blue.

Method	Person	Car	Bus	Truck	Motorcycle	Lamp	All	mAP@.5
Infrared	0.631	0.561	0.544	0.536	0.510	0.512	0.544	0.4354
Visible	0.612	0.591	0.596	0.569	0.547	0.531	0.574	0.4264
DenseFuse	0.633	0.603	0.631	0.583	0.599	0.523	0.595	0.4766
FusionGAN	0.597	0.589	0.584	0.589	0.544	0.534	0.572	0.4784
RFN-Nest	0.613	0.577	0.601	0.576	0.558	0.552	0.580	0.4772
GANMcC	0.582	0.568	0.565	0.510	0.527	0.554	0.551	0.4760
IFCNN	0.506	0.566	0.596	0.566	0.567	0.535	0.556	0.4569
MFEIF	0.622	0.610	0.578	0.576	0.575	0.514	0.579	0.4610
U2Fusion	0.577	0.584	0.596	0.548	0.577	0.558	0.573	0.4642
Ours	0.647	0.607	0.582	0.597	0.583	0.564	0.597	0.4810

**Table 4:** Quantitative results of image fusion in the TNO and Roadscene datasets using different networks. The best result is highlighted in red whereas the second-best one is highlighted in blue.

	Component		TNO Dataset			Roadscene Dataset		
Model	MAAB	NAS	EN	SF	SCD	EN	SF	SCD
M1	×	×	7.310	7.216	1.211	7.365	9.269	1.386
M2	√	×	6.856	9.366	1.365	7.031	12.548	1.753
M3	×	√	7.012	8.998	1.318	7.124	10.945	1.421
M4	√	√	7.186	10.378	1.416	7.341	13.273	1.789

## Data Availability

The program code and data that support the plots discussed within this paper are available from the corresponding author upon request.
